# Pulmonary arterial enlargement predicts cardiopulmonary complications after pulmonary resection for lung cancer: a retrospective cohort study

**DOI:** 10.1186/s13019-015-0315-9

**Published:** 2015-09-09

**Authors:** Keisuke Asakura, Shota Mitsuboshi, Makoto Tsuji, Hiroyuki Sakamaki, Sotaro Otake, Shinsaku Matsuda, Kaoru Kaseda, Kenichi Watanabe

**Affiliations:** 1 Division of Thoracic Surgery, Department of Surgery, School of Medicine, Keio University, 35 Shinanomachi, Shinjuku-ku, Tokyo Japan; 2 Department of General Thoracic Surgery, Sagamihara Kyodo Hospital, Kanagawa, Japan

**Keywords:** Lung cancer, Pulmonary resection, Pulmonary artery, Computed tomography, Pulmonary arterial hypertension

## Abstract

**Background:**

The finding of pulmonary arterial enlargement on computed tomography has been reported to be associated with pulmonary hypertension. On the other hand, pulmonary hypertension is a known risk factor for thoracic surgery. We investigated whether pulmonary arterial enlargement predicts cardiopulmonary complications following pulmonary resection for lung cancer.

**Methods:**

We reviewed 237 consecutive patients who underwent pulmonary resection for lung cancer. Preoperative patient characteristics (sex, age, Brinkman index, cardiopulmonary comorbidities, cardiothoracic ratio, pulmonary function, and pulmonary arterial enlargement) and surgical data (surgical procedure, pathological stage, postoperative complications, mortality, and length of postoperative hospital stay) were analyzed. In order to evaluate preoperative pulmonary arterial enlargement, we measured the diameter of the main pulmonary artery at its bifurcation and that of the ascending aorta at its widest point using chest computed tomography and calculated the ratio of the former diameter to the latter.

**Results:**

In all, 16 patients developed postoperative cardiopulmonary complications and 221 did not. One patient died from postoperative pneumonia. The mean age of patients who developed postoperative cardiopulmonary complications was significantly higher than that of those who did not (78 ± 5 years vs 69 ± 9 years, *P* = 0.0001). The pulmonary artery-to-ascending-aorta ratio was significantly higher in patients who developed postoperative complications than in those who did not (0.94 ± 0.15 vs. 0.81 ± 0.11, *P* = 0.03). Other preoperative patient characteristics and surgical data did not differ significantly between the groups. On multivariate analysis, pulmonary artery-to-ascending-aorta ratio (0.1-point increase; odds ratio 2.3, 95 % confidence interval 1.5–3.5; *P* = 0.0002) and age (1-year increase; odds ratio 1.2, 95 % confidence interval 1.1–1.3; *P* = 0.03) were found to be independent predictors of postoperative cardiopulmonary complications.

**Conclusions:**

A finding of pulmonary arterial enlargement on computed tomography is a potential predictor of postoperative cardiopulmonary complications after lung cancer surgery.

## Background

Surgery is the standard therapeutic option in patients with early-stage lung cancer. Despite advances in surgical techniques and perioperative care, postoperative complication rates remain as high as 23–41 % [1–5]. To improve the safety of surgical treatment for lung cancer, it is important to identify which candidates are at high risk of postoperative complications.

Pulmonary hypertension has been shown to be a predictor of postoperative cardiopulmonary complication (PCC) and mortality after surgery [6–8]. Standard methods for measuring the pulmonary arterial pressure include Doppler echocardiography and cardiac catheterization. However, performing these examinations is not practical in all surgical candidates for reasons of invasiveness and cost. It has been reported that the results of computed tomographic examination for pulmonary arterial enlargement correlate with those of invasive or echocardiographic measurements of pulmonary arterial pressure [9–11]. In the present study, the relationship between a finding of pulmonary arterial enlargement on computed tomography and PCCs following pulmonary resection was retrospectively investigated to clarify the value of pulmonary arterial enlargement as a predictor of PCCs in lung cancer patients.

## Methods

Between October 2008 and May 2013, 241 consecutive patients with primary lung cancer underwent pulmonary resection at Sagamihara Kyodo Hospital, a 400-bed acute-care hospital. For this study, records of 237 of these patients were reviewed retrospectively, with four patients who underwent pneumonectomy being excluded. The Sagamihara Kyodo Hospital Ethical Committee approved the study. In accordance with the Ethical Guidelines for Clinical Studies published by the Japanese Ministry of Health, Labour and Welfare, the Ethical Committee waived the need for individual patients to provide consent because of the retrospective nature of the study, because consent could not be obtained from all of the patients, and because individual patients were not identified in the study.

Preoperatively, all patients underwent computed tomography of the body, magnetic resonance imaging of the brain, ^18^ F-fluoro-2-deoxyglucose positron emission tomography, electrocardiography, and spirometric pulmonary function tests. The spirometric pulmonary function tests included tests of forced expiratory volume in 1 s (FEV_1_) and diffusing capacity of the lung for carbon monoxide (DLCO). Postoperative FEV_1_ and DLCO (ppoFEV_1_ and ppoDLCO) were calculated based on the number of segments to be removed. Data collected preoperatively included the patients’ sex, age, Brinkman index, and cardiopulmonary comorbidities. At our institution, the eligibility criteria for pulmonary resection are a predicted ppoFEV_1_ and ppoDLCO >40 % of the average values in normal individuals (i.e. %ppoFEV_1_ and %ppoDLCO >40). Standard pulmonary resection was performed using video-assisted thoracic surgery with access via two skin incisions: an 8-cm incision used for manipulation and a 1.5-cm incision in which the utility port was placed to allow access for the thoracoscope, staplers, and other instruments.

### Examination for pulmonary arterial enlargement

**Fig. 1:**
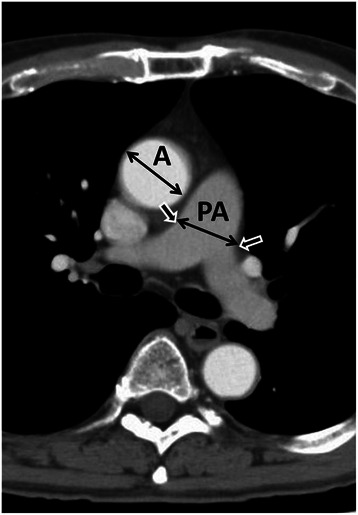
Measurement of the diameters of the pulmonary artery and aorta. Axial chest computed tomographic image at the level of the bifurcation of the main pulmonary artery. Measurements of the diameter of the main pulmonary artery (PA) and that of the aorta (A) at the level of the bifurcation were used to calculate the PA/A ratio. Short arrows show the rises of the bilateral pulmonary arteries. It is the measuring point of the diameter of PA

Axial computed tomography was used to examine the pulmonary artery for enlargement. Measurements were performed by a physician (KA, MT, or SM) who did not know the patient’s postoperative course. In accordance with previous publications [12, 11, 9, 10], we measured the diameter of the main pulmonary artery at the level of its bifurcation and that of the ascending aorta at its widest point, using the same image for both. The measuring point of the main pulmonary artery is at the rises of bilateral pulmonary arteries. The rises of the main pulmonary arteries are obvious on axial computed tomography in most patients, because the angles of outline of pulmonary arteries change sharply at the points (Fig. 1). Then, we calculated the ratio between the diameters (pulmonary artery-to-ascending-aorta ratio: PA/A ratio) as the index of pulmonary arterial enlargement. We also calculated PA/A ratios on computed tomography 3 months after surgery to assess postoperative changes.

### Postoperative cardiopulmonary complications

PCCs of lung resection may include one or more of the following: (1) pneumonia, defined as the appearance of pulmonary infiltrate on radiography, combined with fever and leukocytosis; (2) acute respiratory distress syndrome; (3) respiratory insufficiency requiring tracheostomy; (4) respiratory failure requiring mechanical ventilation; (5) atelectasis requiring bronchoscopic intervention; (6) hypoxaemia requiring home oxygen therapy; (7) arrhythmias requiring medication; (8) coronary artery diseases (angina pectoris and myocardial infarction); (9) acute heart failure requiring medication [13, 14]. Complications related to surgical technique, such as prolonged air leaks, bronchopleural fistulas and chylothorax, were not examined in this study.

### Statistical analyses

Patients were divided into those who developed PCCs and those who developed none, and differences in patient characteristics between the two groups were tested using Fisher’s exact test or the Mann–Whitney *U*-test. The cut-off PA/A ratio most likely to predict the occurrence of PCCs was determined by the receiver operating characteristic (ROC) curve. Among patients who developed PCCs, the incidence of each PCC was compared between those with PA/A ratios above the cut-off value and those with PA/A ratios under it, and differences in PCCs between the two groups were tested using Fisher’s exact test or the Mann–Whitney *U* test. Factors appearing significant on these univariate analyses were further analyzed with multivariate logistic regression to determine if any were independent predictors of the occurrence of PCCs. All tests were two-sided and used a 5 % significance level. All data were analyzed using SPSS software (SPSS Statistics 20, IBM, Armonk, NY, USA).

## Results

### Patient characteristics, surgical data, and postoperative cardiopulmonary complications

Preoperative patient characteristics and surgical data are summarized in Table 1. Of 237 patients who underwent pulmonary resection for lung cancer, 16 patients developed PCCs and 221 did not. Postoperative complications related to surgical procedure (prolonged air leakage in 24 patients and chylothorax in 2 patients) were excluded from the analysis. The mean age of the patients in the PCC group was significantly higher than that of the patients in the non-PCC group (78 ± 5 years vs 69 ± 9 years, *P* = 0.0001). PA/A ratio was significantly higher in the PCC group than in the non-PCC group (0.94 ± 0.15 vs 0.81 ± 0.11, *P* = 0.001). Other preoperative patient characteristics, including sex, cardiothoracic ratio, cardiopulmonary comorbidities, Brinkman index, FEV_1_ as a percentage of the average value in normal individuals (%FEV_1_), ratio of FEV_1_ to forced vital capacity (FEV_1_/FVC), DLCO as a percentage of the average value in normal individuals (%DLCO), predicted %ppoFEV_1_, and predicted %ppoDLCO, did not differ significantly between the two groups. Regarding the surgical procedures, bilobectomy was performed in 3 patients, lobectomy in 182 (including 5 bronchovascular reconstructions and 3 resections of involved neighbouring structures), segmentectomy in 42, and wedge resection in 10. The proportions of patients who underwent lobectomies and other more extensive procedures did not differ significantly between the PCC group and the non-PCC group (*P* = 1). The pathological stage of the disease did not differ significantly between the two groups (*P* = 0.4).

**Table 1:** Preoperative patient characteristics and surgical data

Characteristics	Total	No PCCs	PCCs	
	(n = 237)	(n = 221)	(n = 16)	*P* value
Age, years	70 ± 9	69 ± 9	78 ± 5	0.0001
Male, n (%)	160 (68)	148 (67)	12 (75)	0.6
Cardiopulmonary comorbidity, n (%)	138 (58)	125 (57)	13 (81)	0.07
Cardiothoracic ratio	0.47 ± 0.05	0.47 ± 0.05	0.48 ± 0.08	0.7
Brinkman index	632 ± 627	620 ± 603	801 ± 903	0.6
%FEV_1_	108 ± 24	109 ± 23	100 ± 33	0.2
FEV_1_/FVC	71 ± 11	71 ± 10	68 ± 17	0.7
%DLCO	105 ± 31	105 ± 31	103 ± 31	0.8
%ppoFEV_1_	88 ± 21	87 ± 21	84 ± 29	0.4
%ppoDLCO	85 ± 26	85 ± 26	86 ± 27	0.9
PA/A ratio	0.82 ± 0.12	0.81 ± 0.11	0.90 ± 0.15	0.03
Surgical procedure, n (%)				1
Lobectomy or bilobectomy	185 (78)	172 (78)	13 (81)	
Segmentectomy or wedge resection	52 (22)	49 (22)	3 (19)	
Pathological stage, n (%)				0.4
Stage I	174 (73)	164 (74)	10 (63)	
Stage II or III	63 (27)	57 (26)	6 (38)	

All values are mean ± SD, unless otherwise indicated

*VC* vital capacity, *FEV*_*1*_ forced expiratory volume in 1 s, *FVC* forced vital capacity, *DLCO* diffusing capacity of the lung for carbon monoxide, *ppoFEV1* predicted postoperative forced expiratory volume in 1 s, *ppoDLCO* predicted postoperative diffusing capacity of the lung for carbon monoxide, *PA/A ratio* pulmonary-artery-to-aorta ratio, *POCs* postoperative complications

### Pulmonary arterial enlargement as a predictor of postoperative cardiopulmonary complications

**Fig. 2:**
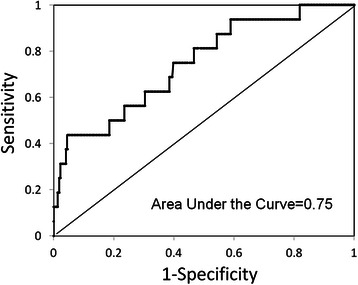
Receiver operating characteristic curves for PA/A ratio in the prediction of postoperative complications. The area under the receiver operating characteristic curve (AUC), a measure of discriminant or predictive power, was 0.75 for the ratio of the pulmonary artery diameter to the aorta diameter. The ratio showed the best combination of sensitivity and specificity for prediction of postoperative complications at values >1.0 (44 % sensitivity and 96 % specificity)

Figure 2 shows the area under the ROC curve (AUC), a measure of discriminant or predictive power, to be 0.75 for PA/A ratio. The ratio showed the best combination of sensitivity and specificity for prediction of PCCs at values >1.0 (44 % sensitivity and 96 % specificity).

### Incidence of particular postoperative cardiopulmonary complications and significance of pulmonary arterial enlargement

Table 2 shows the incidence of particular PCCs. Of the 237 patients in the study sample, 16 developed PCCs for a morbidity rate of 6.8 %. Thirteen patients developed one PCC, and 3 developed two PCCs. Atrial fibrillation occurred in 7 patients, pneumonia in 5, atelectasis in 5, hypoxemia requiring home oxygen therapy in 3, and acute heart failure in 1. The overall incidence of PCCs was significantly higher in patients with a PA/A ratio >1.0 than in those with a PA/A ratio ≤1.0 (35 % vs 4 %, *P* = 0.0001). The incidences of hypoxemia requiring home oxygen therapy was significantly higher in patients with a PA/A ratio >1.0 than in those with a PA/A ratio ≤1.0 (10 % vs 1 %, *P* = 0.02). The incidences of other particular PCCs did not differ significantly between the two groups. One patient with a PA/A ratio ≤1.0 died from pneumonia on postoperative day 129 for an in-hospital mortality rate of 0.4 % (1/241); obviously, the mortality rate did not differ significantly between the two groups (*P* = 1).

**Table 2:** Incidence of postoperative cardiopulmonary complications

PCC	Total	PA/A ratio ≤1.0	PA/A ratio >1.0	*P* value
	(n = 237)	(n =217)	(n = 20)	
Any complication	16 (7)	9 (4)	7 (35)	0.0001
Atrial fibrillation	7 (3)	5 (2)	2 (10)	0.11
Pneumonia	5 (2)	3 (1)	2 (10)	0.06
Atelectasis	5 (2)	3 (1)	2 (10)	0.06
Hypoxaemia	3 (1)	1 (1)	2 (10)	0.02
Acute heart failure	1 (0.4)	0 (0)	1 (0.5)	0.08
30-day mortality	0 (0)	0 (0)	(0)	1.0
In-hospital mortality	1 (0.4)	1 (0.4)	0 (0)	1.0

All values are n (%)

*PCC* postoperative cardiopulmonary complication, *PA/A ratio* pulmonary-artery-to-aorta ratio

**Table 3:** Multivariate analysis of patient characteristics possibly contributing to postoperative cardiopulmonary complications

Variable	Category	Odds ratio	95 % CI	*P* value
PA/A ratio	Lowest	REF	1.5–3.5	0.0002
	0.1-point increase	2.3		
Age (years)	Lowest	REF	1.1–1.3	0.03
	1-year increase	1.2		
Cardiopulmonary comorbidity	-	REF	0.7–11.6	0.2
	+	2.5		

*CI* confidence interval, *PA/A ratio* pulmonary-artery-to-aorta ratio, *REF* reference parameter

### Multivariate analysis

Age (*P* = 0.0001) and PA/A ratio (*P* = 0.03) were significantly different between the PCC and the non-PCC groups on univariate analysis. And cardiopulmonary comorbidities (*P* = 0.07) tended to be more frequent in the PCC group than in the non-PCC group on univariate analysis. These factors were accordingly selected for multivariate logistic regression analysis that identified PA/A ratio (0.1-point increase; odds ratio 2.3, 95 % confidence interval 1.5–3.5; *P* = 0.0002) and age (1-year increase; odds ratio 1.2, 95 % confidence interval 1.1–1.3; *P* = 0.003) as independent predictors of PCCs (Table 3).

### Preoperative and postoperative pulmonary artery size

**Fig. 3:**
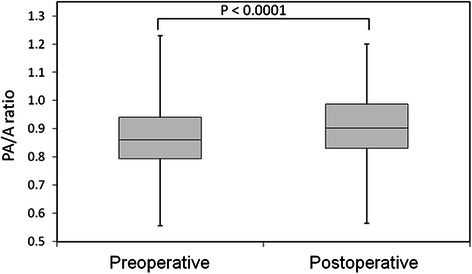
A box-plot showing preoprerative and postoperative PA/A ratio. A box-plot shows the ratio between the diameters of the pulmonary artery and aorta (PA/A ratio) preoperatively and 3 months after surgery. The postoperative PA/A ratio was significantly higher than the preoperative ratio (*P* < 0.0001)

Preoperative and postoperative PA/A ratios are shown in Fig. 3. The postoperative PA/A ratio was significantly higher (median: 0.85; 25th percentile: 0.78; 75th percentile: 0.94) than the preoperative PA/A ratio (median: 0.81; 25th percentile: 0.74, 75th percentile: 0.89) (*P* < 0.0001).

## Discussion

In this study, a finding of pulmonary arterial enlargement on computed tomography was associated with the occurrence of PCCs following lung resection for cancer. Of 237 patients who underwent such surgery, 16 developed PCCs for a morbidity rate of 6.8 %. The incidence of PCCs was significantly higher in patients with PA/A ratios >1.0 than in those with PA/A ratios ≤1.0. Multivariate logistic regression analysis showed that age and PA/A ratio were independent predictors of PCCs.

The present study is the first to show the relationship between pulmonary arterial enlargement on computed tomography and the occurrence of PCCs following lung resection for cancer. It is well known that pulmonary arterial hypertension is associated with various diseases, including left-sided heart disease, chronic obstructive pulmonary disease, interstitial pulmonary disorders, and pulmonary vascular disorders [15, 11, 16]. Moreover, several studies indicate that preoperative pulmonary hypertension may be a predictor of PCCs [7, 6, 8]. As we did not routinely perform Doppler echocardiography, we could not analyze the relationship between PA/A ratio and pulmonary arterial pressure in our patients. However, several previous studies have demonstrated that measurements of pulmonary arterial enlargement based on computed tomography correlate with invasive or echocardiographic measurements of pulmonary arterial pressure [9–11]. Iyer et al. reported that a PA/A ratio >1.0 was 73 % sensitive and 84 % specific in identifying patients with mean pulmonary arterial pressure >25 mmHg [11]. This suggests that the high incidence of PCCs in patients with PA/A ratios >1.0 in the present study was caused, at least in part, by pulmonary arterial hypertension. Why is pulmonary hypertension in lung resection patients associated with PCCs? One hypothesis is that pulmonary resection causes the loss of the pulmonary artery bed, which elevates pulmonary arterial pressure and may result in right ventricular dysfunction, particularly in patients who already had pulmonary hypertension preoperatively [17]. Indeed, the current study found PA/A ratios 3 months after surgery to be significantly higher than those before surgery.

Although FEV_1_ [18, 2] and DLCO [3, 2] have previously been reported to be strongly predictive of PCCs, they were not significant predictors of PCCs in our study, possibly because we excluded patients with very poor pulmonary function from surgery. In fact, the predicted postoperative pulmonary functions in our patients were very good, with a mean predicted %ppoFEV_1_ of 87 % ± 22 % and a mean predicted %ppoDLCO of 85 % ± 26 %.

The calculation of PA/A ratio based on computed tomography is a well-established method for detecting pulmonary hypertension [12, 11, 16, 9, 10]. Measuring the diameter of main pulmonary artery at the rises of bilateral pulmonary arteries is considered as a reproducible method. Iyer et al. reported the Cohen kappa of 0.82 (95 % CI, 0.68-0.97) for interobserver agreement in the PA/A ratio based on computed tomography. A Cohen’s Kappa above 0.8 is regarded as almost perfect agreement. Additionally, the method involves no additional cost and is non-invasive, as all candidates for lung resection undergo chest computed tomography preoperatively. Furthermore, the calculation can be completed in approximately 30 s. Given these advantages, PA/A ratio is recommended for use in the assessment of candidates for pulmonary resection.

This study had several limitations. Firstly, this study was limited by its retrospective design. Secondly, the number of patients involved in the study was small (n = 241). The sample was of similar size to samples in several recent reports from other organizations investigating predictive factors for PCCs following surgery for lung cancer [14, 5, 19]. However the morbidity of PCCs was very low (6.7 %, n = 16) in the present study, so the number of patients might be inadequate to evaluate the relationship between and PA/A ratio and each particular PCCs. Thirdly, we could not analyze the relationship between PA/A ratio and pulmonary arterial pressure, as we did not routinely perform Doppler echocardiography. Fourthly, cardiopulmonary exercise testing is currently considered the gold standard of risk assessment for lung resection candidates [20]; however, few of the patients in our sample underwent such testing. To further clarify the clinical significance of PA/A ratio in pulmonary resection candidates, large-scale prospective studies investigating its relationships with pulmonary arterial pressure and cardiopulmonary exercise testing are needed.

## Conclusions

A finding of pulmonary arterial enlargement on computed tomography (PA/A ratio >1.0) is an independent predictor of PCCs following pulmonary resection for lung cancer in this particular study. Further investigation on PA/A ratio in a large-scale prospective study is needed.

## Abbreviations

PCC: postoperative cardiopulmonary complication
FEV_1_: forced expiratory volume in 1 s
DLCO: diffusing capacity of the lung for carbon monoxide
ppoFEV_1_: postoperative forced expiratory volume in 1 s
ppoDLCO: postoperative diffusing capacity of the lung for carbon monoxide
ROC: receiver operating characteristic
